# Endoscopic band ligation for weight loss: a case report with 6-month follow-up

**DOI:** 10.1016/j.igie.2025.10.003

**Published:** 2025-10-14

**Authors:** Mohamed Abeid

**Affiliations:** Faculty of Medicine, Cairo University, Giza, Egypt

## Abstract

Endoscopic band ligation (EBL) is an established treatment for variceal bleeding and gastric antral vascular ectasia. We report its novel application for weight loss in a 47-year-old woman with morbid obesity (body mass index 55 kg/m^2^, weight 140 kg) who had declined surgery and pharmacotherapy. The patient underwent EBL under propofol sedation with 15 bands placed in the gastric antrum using a standard multiband ligator. The procedure lasted 12 minutes. Early satiety and appetite suppression were reported. At 6 months, weight decreased to 90 kg (body mass index 35), with 36% total weight loss and 67% excess weight loss. Endoscopy confirmed healing of postprocedure ulcers and marginal antral narrowing. No adverse events occurred. EBL appears to be a promising, safe, and cost-effective endoscopic technique for weight loss. Further studies are needed to validate its efficacy and explore underlying mechanisms.

## Introduction

Obesity is a leading cause of global morbidity and mortality. Despite the efficacy of bariatric surgery and pharmacotherapy, their widespread use is restricted by high costs, potential adverse events, and limited accessibility. Endoscopic band ligation (EBL), a technique widely used for the management of esophageal varices^1^ and gastric antral vascular ectasia,^2^ has demonstrated an excellent safety profile. Here, we report a case highlighting EBL as a minimally invasive, cost-effective weight loss option. This article is distinct from our previously published clinical trials,^3^^,^^4^ which focused on EBL in the gastric body. To our knowledge, this case represents the first documented application of EBL in the gastric antrum with a comprehensive 6-month follow-up.

## Case presentation

A 47-year-old woman with morbid obesity (body mass index [BMI] 55 kg/m^2^, weight 140 kg) had multiple unsuccessful conservative weight loss attempts and declined surgery and pharmacotherapy because of concerns over safety and affordability. There was no significant family history of obesity, endocrine disorders, or metabolic disease. Social history was unremarkable. On physical examination, the patient was hemodynamically stable, with no signs of acute distress. Findings of cardiopulmonary and abdominal examinations were unremarkable.

The procedure adhered to the Declaration of Helsinki and was registered at ClinicalTrials.gov (NCT04431232). With the patient under propofol sedation, 15 bands were randomly deployed in the gastric antrum using a standard multiband ligator (Video 1, available online at www.igiejournal.org). The 12-minute procedure was completed without any adverse events (Fig. 1). The patient was discharged 2 hours later in stable condition. A follow-up endoscopy performed 2 weeks after the procedure revealed multiple postband ulcers and early signs of antral narrowing (Fig. 2).

**Figure 1:**
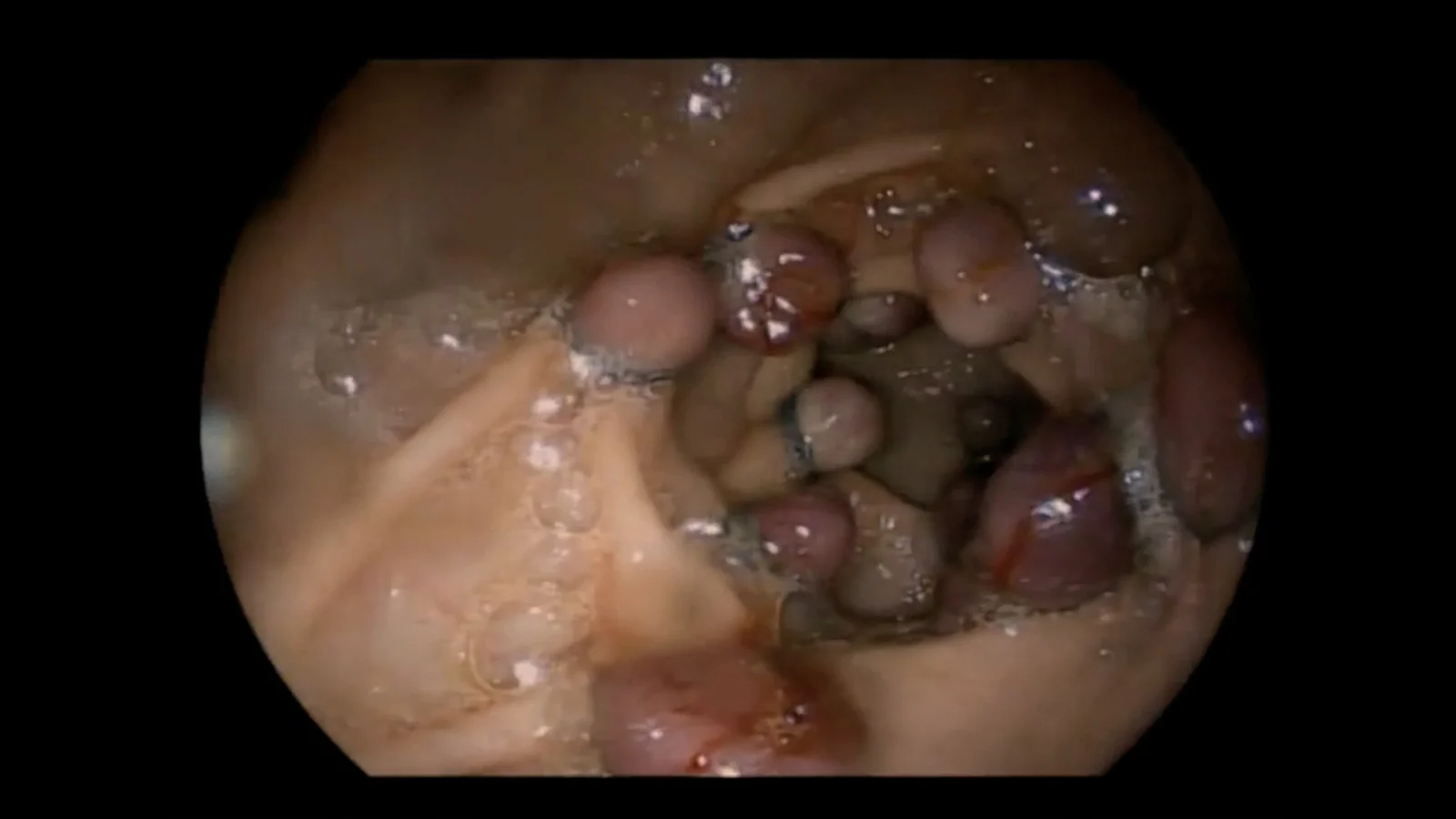
Band ligation in the gastric antrum.

**Figure 2:**
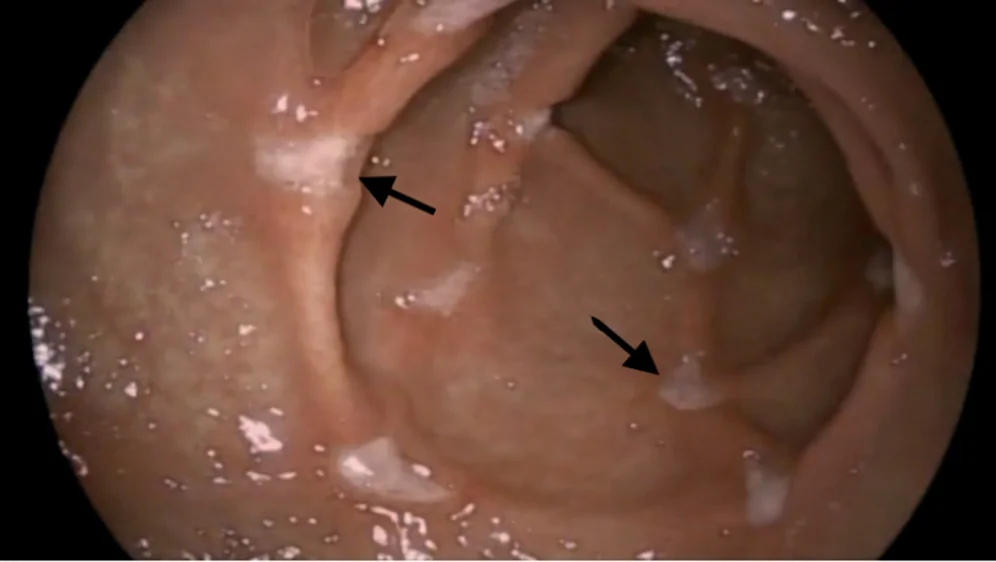
Follow-up endoscopy 2 weeks later showing multiple postband ulcers (*arrows*).

The patient experienced mild nausea and epigastric pain during the first 48 hours, which was managed conservatively. A staged bariatric-style diet was followed: a liquid diet for 2 weeks, followed by pureed foods for 2 weeks, then a 1200-calorie solid diet starting in the second month. Early satiety and prolonged satiation were reported from day 1. The patient also was advised to walk 30 minutes daily.

At 3-month follow-up, weight was 110 kg (BMI 43 kg/m^2^), with 21% total weight loss and 39% excess weight loss. Gastroparesis Cardinal Symptom Index (GCSI) scores were: nausea 0, fullness 3, and bloating 0. Endoscopy showed healed ulcers and marginal antral narrowing (Fig. 3). At 6 months, weight reached 90 kg (BMI 35 kg/m^2^), total weight loss was 36%, and excess weight loss 67%. GCSI remained unchanged. Weight loss over time is illustrated in Figure 4. Baseline GCSI was presumed to be 0, because the patient did not report symptoms of nausea, bloating, or early satiety before the procedure. The patient expressed high satisfaction with the results of the procedure, reporting significant improvements in quality of life, mobility, and self-confidence after the substantial weight loss.

**Figure 3:**
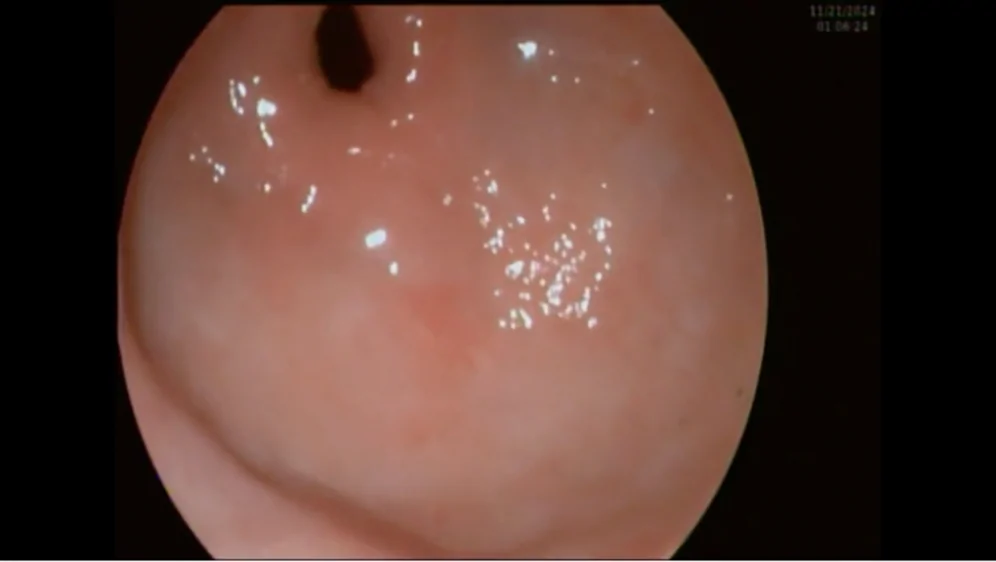
Follow-up endoscopy 3 months later showing complete healing with marginal narrowing of the lumen.

**Figure 4:**
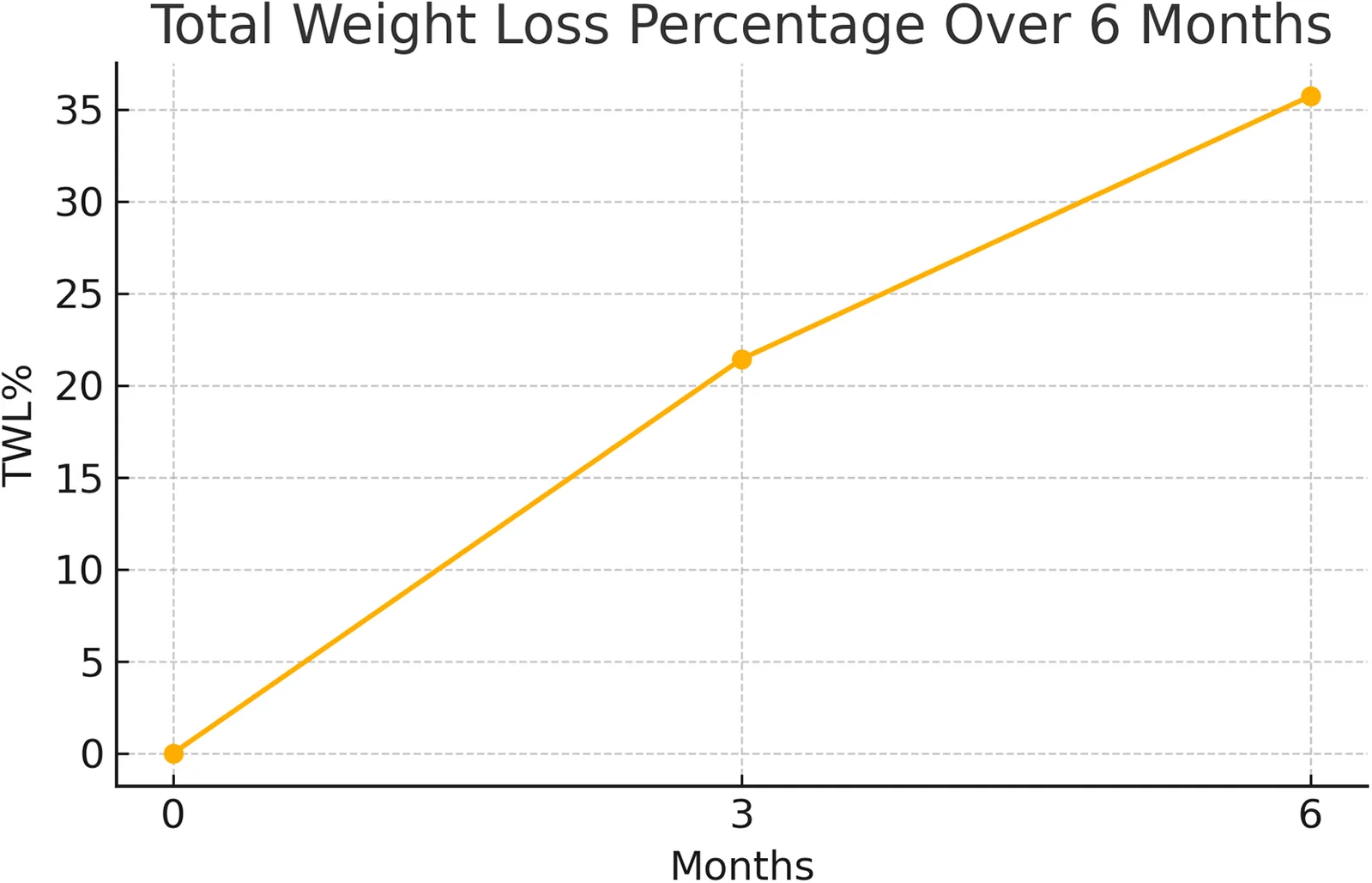
Graph showing total weight loss percentage (TWL%) over 6 months.

## Discussion

EBL may induce weight loss through mechanical narrowing of the gastric lumen, delayed gastric emptying, reduced antral motility, and hormonal changes such as ghrelin suppression.^3^^,^^4^ These proposed mechanisms remain theoretical and require confirmation through larger controlled studies. This case demonstrates significant weight reduction with no severe adverse events. No antiobesity or off-label weight loss medications were used before or after the procedure. EBL requires minimal additional training and equipment, making it particularly attractive in low-resource settings. Unlike previous reports that targeted the gastric body, this case uniquely demonstrates the safety and efficacy of banding in the gastric antrum. To our knowledge, it is the first published report focusing exclusively on this anatomical site. The 36% total weight loss seen here greatly exceeds the 7.8% total weight loss we previously reported at 1 month following EBL in the gastric body.^4^ This suggests that the current patient may be a “super responder,” and such outcomes may not be generalizable to all individuals undergoing this technique. Limitations of this case include lack of baseline GCSI documentation, reliance on subjective appetite reporting, and limited generalizability given the single-patient design. Further controlled studies are needed.

## Conclusions

EBL of the gastric antrum appears to be a safe, effective, and affordable approach for obesity management. Larger controlled trials are needed to confirm these preliminary findings.

## Declaration of generative artificial intelligence and artificial intelligence–assisted technologies in the writing process

During the preparation of this work, the author used ChatGPT (OpenAI, San Francisco, Calif, USA) to improve language, structure, and readability. After using this tool, the author reviewed and edited the content as needed and takes full responsibility for the content of the publication.

## Patient consent statement

Written informed consent was obtained from the patient for publication of this case report and any accompanying images and video.

## Disclosure

The author disclosed no financial relationships.

## Supplementary data

Video 1 Endoscopic band ligation for weight loss (available online).

## References

[1] Gimson A.E., Ramage J.K., Panos MZ. (1993). Randomised trial of variceal banding ligation versus injection sclerotherapy for bleeding oesophageal varices. Lancet.

[2] Mohan BP., Toy G, Kassab LL (2021). Endoscopic band ligation in the treatment of gastric antral vascular ectasia: a systematic review and meta-analysis. Gastrointest Endosc.

[3] Abeid M., Kaddah T. (2021). Endoscopic band ligation for weight loss. Endoscopy.

[4] Abeid M., Zaitoun N. (2025). Endoscopic band ligation for weight loss: a clinical trial. Obes Surg.

